# The Heterogeneity of Mental Health Assessment

**DOI:** 10.3389/fpsyt.2020.00076

**Published:** 2020-02-27

**Authors:** Jennifer J. Newson, Daniel Hunter, Tara C. Thiagarajan

**Affiliations:** Sapien Labs, Arlington, VA, United States

**Keywords:** mental health, psychiatric, diagnosis, transdiagnostic, questionnaires, assessment, cross-disorder

## Abstract

Across the landscape of mental health research and diagnosis, there is a diverse range of questionnaires and interviews available for use by clinicians and researchers to determine patient treatment plans or investigate internal and external etiologies. Although individually, these tools have each been assessed for their validity and reliability, there is little research examining the consistency between them in terms of what symptoms they assess, and how they assess those symptoms. Here, we provide an analysis of 126 different questionnaires and interviews commonly used to diagnose and screen for 10 different disorder types including depression, anxiety, obsessive compulsive disorder (OCD), post-traumatic stress disorder (PTSD), attention deficit/hyperactivity disorder (ADHD), autism spectrum disorder (ASD), addiction, bipolar disorder, eating disorder, and schizophrenia, as well as comparator questionnaires and interviews that offer an all-in-one cross-disorder assessment of mental health. We demonstrate substantial inconsistency in the inclusion and emphasis of symptoms assessed within disorders as well as considerable symptom overlap across disorder-specific tools. Within the same disorder, similarity scores across assessment tools ranged from 29% for assessment of bipolar disorder to a maximum of 58% for OCD. Furthermore, when looking across disorders, 60% of symptoms were assessed in at least half of all disorders illustrating the extensive overlap in symptom profiles between disorder-specific assessment tools. Biases in assessment toward emotional, cognitive, physical or behavioral symptoms were also observed, further adding to the heterogeneity across assessments. Analysis of other characteristics such as the time period over which symptoms were assessed, as well as whether there was a focus toward frequency, severity or duration of symptoms also varied substantially across assessment tools. The consequence of this inconsistent and heterogeneous assessment landscape is that it hinders clinical diagnosis and treatment and frustrates understanding of the social, environmental, and biological factors that contribute to mental health symptoms and disorders. Altogether, it underscores the need for standardized assessment tools that are more disorder agnostic and span the full spectrum of mental health symptoms to aid the understanding of underlying etiologies and the discovery of new treatments for psychiatric dysfunction.

## Introduction

Across clinical and research domains, mental health assessment and diagnosis are carried out using interviews and questionnaires that determine the presence, severity, frequency, and duration of a broad range of psychiatric symptoms. The question content of these assessment tools is often based on classification systems such as the Diagnostic and Statistical Manual of Mental Disorders (DSM-5) (1) or International Classification of Diseases (ICD-11) (2) where pre-defined patterns of symptom criteria have been grouped together and designated as specific mental health disorders. Their design ranges from more open-ended clinician-led interviews typically used to make a formal psychiatric diagnosis (e.g., SCID) (3), to more quantitatively designed auxiliary questionnaires (e.g., PHQ-9) (4) that provide multidimensional assessments of symptom experience and severity to support diagnosis and treatment evaluation in clinical practice, and that are used to investigate underlying etiologies and treatment effectiveness in clinical trials and academic research studies.

A top down perspective of this landscape of mental health assessment reveals a huge range of interviews and questionnaires available for use. This diversity of choice means there is no shortage of options when searching for assessment tools for clinical use or to suit the needs of a clinical research study. However, this diversity can also make it a real challenge to decide which questionnaire(s) or interview(s) to select for clinical diagnosis or evaluation. For example, there have been more than 280 different questionnaires developed over the last century to assess symptoms of depression (5) which differ in terms of which iteration of the DSM they align to, the degree to which they consider co-morbid symptoms, whether they are computer based or paper based, and whether they are self-rated, parent-rated or clinician-led. Knowing which questionnaire to choose to obtain a suitable assessment of an individual’s mental health is therefore not always a straightforward exercise for even the most experienced researcher or clinician.

The significance of having so many assessment options is two-fold. Firstly, within the clinic, screening questionnaires and interviews allow clinicians to build up a picture of the psychological problems and concerns faced by their patients so they can determine a diagnosis that will then determine a treatment regime. However, when different clinicians use different assessment tools during patient evaluations, it has the potential to introduce variability and inconsistency in diagnosis (6). Furthermore, as many assessment and diagnostic tools are disorder specific, a patient’s experience rarely fits neatly within these theoretically defined boundaries, and therefore the choice of tool can potentially bias diagnosis. In addition, the onset and trajectory of a mental health disorder is often impacted by numerous social and environmental factors. If clinicians use assessment tools that differ by the degree to which they ask about these factors (e.g., triggers), especially for disorders where external events are known to have a considerable impact (e.g., depression), then it may provide a varying or incomplete picture of these contributing elements, which in turn could hinder the delivery of appropriate patient treatment plans, or provide conflicting evidence in relation to mental health recommendations and policies.

Secondly, across the landscape of mental health research there is a drive to better understand underlying etiologies to help deliver new treatment opportunities. One avenue is the development of physiological biomarkers to aid diagnosis of mental health disorders and guide discovery of pharmacological intervention (7–12), although see (13, 14). These studies often aim to reveal correlations between neurobiological changes in a patient’s brain (e.g., using tools such as fMRI and EEG) and the presence or severity of a mental health diagnosis. However, when these studies use different questionnaires and interviews to review the characteristics and severity of a patient’s thoughts, feelings, and actions, it has the potential to introduce considerable variability that hinders comparisons across studies and reproducibility. Similarly, when researchers use different questionnaires or interviews in the assessment of symptom severity in studies evaluating the effectiveness of new or existing pharmacological, cognitive and/or behavioral therapies, or when trying to understand psychopathological mechanisms or the phenomenology of a disorder, it again has the potential to introduce variability and impede attempts to demonstrate the validity and reliability of results across studies.

Despite the large variety of assessment and diagnostic tools, there is relatively little research that has provided an in-depth analysis of the consistency of symptoms assessed across questionnaires both within and between different mental health disorders [although see (15) for a within disorder perspective of depression]. For the purposes of this paper, the term consistency denotes the degree to which different assessment tools designed for the same disorder assess the same set of symptoms, and whether they assess those symptoms using similar questioning characteristics. By identifying the level of consistency, or inconsistency, across assessment tools it allows us to determine the true extent of the potential problems noted above, and also allows researchers and clinicians to see how their favored choice of assessment tool(s) could be influencing or biasing their assessment or results. For example, if one clinician preferably uses a tool that predominantly assesses behaviors and actions, while another uses a tool (developed for the same disorder) that focuses on emotional difficulties, then the clinicians may end up creating different clinical impressions of the patients in front of them.

To explore this, we carried out an analysis of 126 questionnaires and interviews used to diagnose and screen for 10 different disorder types, including depression, anxiety, obsessive compulsive disorder (OCD), post-traumatic stress disorder (PTSD), attention deficit/hyperactivity disorder (ADHD), autism spectrum disorder (ASD), addiction (drug and alcohol), bipolar disorder, eating disorder, and schizophrenia, as well as comparator questionnaires and interviews that offer an all-in-one cross-disorder assessment of mental health. These cross-disorder tools were included to provide a perspective on the breadth of symptoms assessed by each tool to reveal which ones covered the widest spectrum of mental health symptoms, as well as to determine the consistency of symptoms assessed by these tools.

As this analysis was conducted purely on the written content of the questions within an assessment or diagnostic tool, rather than on responses made by any participant group, our aim was not to determine the reliability or consistency of responses to individual questionnaires, nor was it to cluster symptoms according to patient responses to these questionnaires. Instead, our objective was to investigate the range and diversity of symptoms assessed across these tools as well as the way those symptoms were assessed, to explore any potential inconsistences in the landscape of mental health assessment. In particular, we hypothesized that screening tools within individual disorders would be highly consistent across core sets of symptoms, revealing standardized diagnostic criteria. On the other hand, we hypothesized that while there would also be some overlap in the symptoms assessed across disorders, individual disorders would be relatively distinct in symptom profile. In contrast to this *a priori* hypothesis we found a highly inconsistent and heterogeneous landscape within and across disorders. The specific set of symptoms assessed within disorders, as well as the degree to which assessments focused on cognitive, emotional, physical or behavioral symptoms, and the extent to which they asked about symptom triggers or consequences, varied dramatically as did assessments of symptom time frame, and whether they focused on the presence, frequency, duration or timing of symptoms. Furthermore, most symptoms were very broadly distributed across multiple disorders rather than being disorder specific. We also hypothesized that cross-disorder screening tools would cover a wide breadth of symptoms that spanned symptoms across all major mental health disorders with a high degree of similarity. However, we found the opposite, where cross-disorder tools were generally incomplete and differed in the breadth of symptoms they asked about as well as the degree to which they focused on emotional, behavioral, physical, and cognitive symptoms.

## Materials and Methods

We conducted an analysis of clinical interviews and screening questionnaires commonly used within a clinical or research domain. The analysis focused on 10 psychiatric disorders including depression, anxiety, OCD, PTSD ADHD, ASD, addiction, bipolar disorder, eating disorder and schizophrenia, covering questionnaires and interviews developed for adult, and pediatric populations. These disorders were selected based on a review of the disorders included in the DSM clinical interview (SCID-CV) (3). In addition, ASD and eating disorder were included due to both their prevalence and their broad public and scientific interest. Also included are a number of commonly used questionnaires and interviews which were not specific to any one disorder, but which instead took a cross-disorder approach to mental health assessment.

### Coding and Analysis

The study was conducted in three steps. First, we identified and selected a comprehensive set of psychiatric questionnaires and clinical interviews that were commonly cited in the scientific literature. Next, we systematically categorized the individual questions included in each of the selected questionnaires by symptoms and themes. Finally, we compared the symptoms assessed across questionnaires and interviews, both within individual disorder types, and from a cross-disorder perspective, computing similarity scores across all.

#### Questionnaires Identification and Selection

To select the questionnaires, we conducted several different searches and reviews of the literature. Firstly, we conducted a search of PubMed^1^ (November 2019) using combinations of the following keywords in the title “questionnaire OR interview OR rating OR scale OR inventory OR instrument OR measure” alongside the key terms for each disorder of interest. This revealed a total of 10,318 search entries, from which the names of 929 different assessment tools were extracted. Secondly, we collated together a set of meta-analyses and reviews which specifically compared different assessment methodologies for each of the 10 disorders and identified the common questionnaires and interviews reviewed in those publications. Thirdly, we reviewed a number of websites and publishers which included lists of psychiatric assessment tools. Finally, we went through a large-scale cross-disorder review which covered 9 out of the 10 psychiatric disorders included in this review (16). Altogether, these searches revealed a large number of different screening questionnaires and clinical interviews.

To narrow down this larger group of questionnaires and interviews, we first identified the primary citation reference for each assessment tool and then identified the number of Google Scholar citations for each as a measure of use in clinical and academic research. We then excluded assessment tools based on the following criteria: (i) those that specifically covered multiple disorders (e.g., assessed anxiety and depression together); (ii) those that only assessed a specific subset of symptoms associated with a disorder (e.g., only cognitive aspects); (iii) those that only targeted a specific clinical group (e.g., stroke patients); (iv) those that had been used to assess a disorder but weren’t specific to that disorder (e.g., when an OCD tool is used to assess symptoms of schizophrenia); (v) those which were not specific to any one disorder (e.g., assessing medication compliance). We then selected the questionnaires and interviews with higher citation numbers, while also taking into account their publication date. Although we acknowledge that the citation numbers provided by Google Scholar are often higher than those provided from other sources (e.g., Web of Science) we considered this approach to be acceptable when comparing between references. Where accurate citation numbers were not readily available from Google Scholar (e.g., if the questionnaire was reported in a manual or book) then we made a judgement according to how frequently the tool was mentioned/used across the studies we reviewed as to whether it should be included within our final analysis. The final list of questionnaires and interviews we have included is by no means exhaustive but aims to include as many of the most common tools used within clinical and research settings as possible.

Altogether 126 questionnaires and clinical interviews published between 1959 and 2018 were selected for inclusion in the final analysis (**Table 1**, **Supplementary Table 1**) that together had a total of 10,154 questions. Where there were updated versions of a questionnaire, the latest or revised version of a questionnaire or interview was used although older versions were included if they were still commonly used. Assessment tools were obtained from the relevant publisher, publication or author and permission was sought where necessary. Of these assessment tools 82 were specifically designed for adults and 44 were specifically designed for pediatric populations. However, it is important to note that the adolescent age range falls within the adult range for some assessment tools, but within the pediatric range for others, depending on the specific questionnaire or interview. Where multiple versions of a questionnaire or interview were available, we selected the self-rated version. This was done to reflect the dominant prevalence of self-report tools in the mental health assessment literature, especially in relation to auxiliary tools, so as to strengthen comparisons between tools. Exceptions to this were for ASD and ADHD pediatric scales where the parent version was typically selected as this was the most commonly used approach for these disorders.

**Table 1:** Overview of Questionnaires and Interviews included in the analysis.

Disorder	Number of Questionnaires	Number of questions reviewed	List of Assessment Tools Included*
Depression	19	369	*Adult:* APA-Dep-A^1^, BDI-II (17), CES-D (18), CESDR (19), EPDS (20), GDS-LF (21), GDS-SF (22), HAMD (23), IDS (24), MADRS (25), PHQ9 (4), QIDS (26), ZDS (27). *Pediatric:* APA-Dep-C^1^, CDI2 (28, 29), CESDC (30), MFQ (31), MFQS (31), RADS2 (32).
Anxiety	13**	483	*Adult:* APA-Anx-A^1^**, BAI (33), GAD7 (34), HAMA (35), LSAS (36, 37), SPIN (38), STAI (39), ZAS (40). *Pediatric:* APA-Anx-C^1^**, MASC (41, 42), RCMAS (43), SCARED (44, 45), SCAS (46).
PTSD	9	376	*Adult:* APA-PTSD-A^1^, CAPS-5 (47), PC-PTSD-5 (48), PCL-5 (49), PSS-SR5 (50), SPRINT (51). *Pediatric:* APA-PTSD-C^1^, CAPS-5-CV (52), CPSS-V (53).
Bipolar/Mania	5	90	*Adult:* ASRMS (54), HCL32 (55), ISS (56), MDQ (57), YMS (58).
OCD	8	330	*Adult:* DOCS (59), FOCI ™ (60), OCI-R (61), PI-WSUR (62), VOCI (63), Y-BOCS ™ (64, 65). *Pediatric:* CY-BOCS™ (66), OCI-CV (67).
Addiction	13	319	*Adult:* ADS (68, 69), ASI-5 (70), ASSIST-3 (71), AUDIT (72), CAGE (73), DAST-10 (74), DAST-20 (74), DUDIT (75), MAST (76), OCDS (77), SMAST (78), TWEAK (79). *Pediatric:* CRAFFT (80).
ASD	22	1213	*Adult:* AAA (81), AQ-A-10 (82), AQ-A (83), BAPQ (84), EQ-A (85), SQ-A (86). *Pediatric:* ADI-R (87, 88), AQ-Adol-10 (82), AQ-Adol (89), AQ-C-10 (82), AQ-C (90), ASSQ (91), CARS2-HF (92, 93), CARS2-ST (92, 93), CAST (94), EQ-Adol (95), EQSQ (96), GARS-3 (97), M-Chat (98, 99), SCQ (100), SQ-Adol (95), SRS2 (101).
ADHD	9	418	*Adult:* ASRS-5 (102), ASRS-Checklist (103), CAARS (104), DIVA 2.0 (105), WURS (106). *Pediatric:* APA Inatt^1^, Conners 3 ™ (107), DBDRS (108), NICHQ (109).
Schizophrenia	6	136	*Adult:* BPRS (110, 111), CGI-SCH (112), NSA-16 (113), PANSS (114), SANS (115–117), SAPS (118),
Eating Disorder	6	230	BITE (119), EAT-26 (120), EDDS (121), EDE-Q (122), EDI-3 (123), SCOFF (124).
Cross-Category	16	6190	*Adult:* APA-CC-A^1^, BSI (125), CIDI CAPI (126, 127), K10+ (128), MINI (129), PROMIS-Profile-A (130, 131), PROMIS-QB-A (130, 131), SCID-5-CV (3), SCL-90-R (132). *Pediatric:* APA-CC-C^1^, CBC (133), DISC-IV (134), KSADS-PL-5 (135) PROMIS-Profile-C (130, 131), PROMIS-QB-C (130, 131), SDS (136).

*See **Supplementary Table 1** for further details and **Supplementary Table 5** for a list of abbreviations.

**includes multiple APA anxiety assessments – see **Supplementary Table 1.**

^1^ https://www.psychiatry.org/psychiatrists/practice/dsm/educational-resources/assessment-measures.

**Table 1** provides a summary of the specific questionnaires included along with the total number of questions (see **Supplementary Table 5** for a list of abbreviations). The largest number of questionnaires or questions for a single disorder were for ASD followed by anxiety and depression.

#### Question Coding According to Symptom Category and Theme

Once we had selected these questionnaires and interviews, we then performed a systematic coding of each question within each questionnaire or interview to reflect the symptom(s) that each individual question assessed. This was done in two ways as described below.

##### Symptom Category Coding and Analysis

Firstly, the symptom(s) assessed in each individual question was identified and coded based on a judgement of the semantic content of the question. This resulted in 170 different preliminary symptom codings. For example, if a question asked about a patient’s difficulties with concentration or keeping focus on one thing, then the question was coded with the preliminary symptom coding of “difficulty concentrating”, while if a question asked about difficulties falling asleep or about insomnia then it was coded with the preliminary symptom coding of “sleep problems”.

These 170 preliminary symptom codings were then reviewed across all 10,154 questions and consolidated into a set of 43 master symptom categories which were used for the final analysis. This consolidation was performed by grouping together similar preliminary symptom codings, where those judged as being related were assigned to the same symptom category (for example, “concentration difficulties”, “being easily distracted”, “focusing on the big picture”, and “mental fog” were all assigned to the category of “Attention, Concentration & Mental Focus”). The mapping of different preliminary codings to the symptom categories is provided in **Supplementary Table 2**.

The consolidation of the preliminary symptom codings into the 43 symptom categories required the grouping together of related symptoms into a single category. The choice to use a smaller number of symptom categories, rather than selecting all 170 preliminary symptom codings was both a practical one, and one which aimed to ensure our analysis didn’t result in a high level of false positives where dissimilarities were over-emphasized. In this context, there may be some debate about the placement of a particular symptom within a common symptom category, based on an interpretation of their independence. While we consider the 43 symptom categories to be sufficiently distinct from one another in terms of the symptoms that they reflect, they may nonetheless arise from a common underlying cause in the same way that a fever and headache may arise from the same underlying etiology. Conversely, it is possible that similar symptoms grouped in the same symptom category may be interpreted in distinct ways by the respondent (for example “mental fog” and “difficulty concentrating”) or arise from distinct causes. However, given that the underlying etiologies and relationships are not well established, mental health assessment, in general, arises at the level of psychological symptoms that are subject to semantic interpretation. By grouping together related symptoms, we believe we are more likely to have analyzed independent symptoms as being similar, rather than have analyzed similar symptoms as being dissimilar.

Within the dataset, 1.7% of questions (129) were marked as “excluded” because they either related to general functioning or to functions which were not specifically related to mental health and were not included in the analysis. For example, questions that asked about employment, hobbies or marital status were marked as “excluded”, while questions which asked about general concerns, such as “What concerns you most about your child”, were also excluded as they did not fit into any one symptom category. In addition, several of the cross-disorder tools were composed of multiple modules, with each module pertaining to a different disorder. In these instances, only modules which were related to the 10 clinical disorders of interest were included in the analysis and modules which specifically focused on other disorders were excluded (see **Supplementary Table 1** for a list of included modules for the relevant interview assessments). This was done for practical reasons to focus the scope of the analysis towards the 10 disorders of interest. Where cross-disorder tools were not divided up into modules, all questions were included in the analysis. In addition, although the hierarchical nature of clinical questioning means that some questions within an interview are optional for the clinician (i.e., follow up questioning is dependent on the initial responses given by the patient), all interview questions were coded. Furthermore, while it may the case that some questions within a questionnaire or interview do not contribute to a scoring algorithm or directly align with the symptom criteria which lead towards a formal diagnosis, they none the less would likely guide the clinician or researcher during the assessment and so all questions were included in the coding.

The coding of symptom category for each individual question was done in the following way: One researcher conducted an initial coding of all the questions by determining a preliminary symptom coding for each question that was then assigned to one of the 43 symptom categories as described above. A second researcher then reviewed, unblinded, these symptom category codings, across all 10,154 questions, and stated whether they agreed or disagreed with each coding. If they disagreed with a category coding for a particular question, they then generated an alternative coding. For each question where there was a disagreement in the coding between the two researchers, the difference was discussed between them, as well as reviewed by a third researcher to decide on the most appropriate coding for that question. This reassessment process was required for 0.3% of the questions.

For each questionnaire or interview, the proportion of questions corresponding to each of the 43 symptom categories was calculated. These counts were then converted into percentages to take into account the overall number of questions in that questionnaire or interview. This meant that the individual assessment tools could be more easily compared with one another without being biased by the fact that some assessment tools contained more questions than others. These percentages were then used in analysis of symptom similarity (see *Symptom Similarity Analysis*) and also averaged across the questionnaires and interviews corresponding to a particular disorder (e.g., all the ASD questionnaires and interviews) to provide aggregate views of the distribution of symptoms across each of the 10 disorders, as well as for the cross-disorder tools (**Figures 4B, C**).

##### Symptom Theme Coding and Analysis

The questions were then each coded according to the following “themes” representing the particular aspect of the symptom that was assessed: emotion (a feeling), cognitive (a thought), behavior (a behavior or action), physical (a physical or bodily symptom), trigger (the cause of a symptom), consequence (the outcome of a symptom), or treatment (medication or medical treatment relating to that symptom). The systematic coding of symptom themes was based on a semantic judgement of the content of the questions.

In this case both single and dual codings were used. Single coded items were used when questions asked only about one symptom theme (e.g., how someone felt; whether they experienced a particular physical symptom). Dual codings were used to reflect the fact that some questions encompassed multiple themes such as a trigger and an emotion; a behavior and a thought. For example, a question that asked whether someone “tried hard not to think about it or went out of their way to avoid it” was dual coded as cognitive/behavior, while a question that stated “I have crying spells or feel like it” was dual coded as behavior/emotion. In addition, some symptoms more intuitively reflected dual themes. For example, “distressing flashbacks of unwanted memories” includes both emotion and cognitive elements and therefore were dual coded as emotion/cognitive. Similarly, “having a conversation” has both cognitive and behavioral elements and was dual coded as cognitive/behavior. This resulted in 30% of all questions being assigned a dual coding.

The coding of symptom themes was also carried out by two independent researchers in the same way as described above for symptom categories. For each question where there was a disagreement in the coding between the two researchers, the difference was reviewed between them, as well as by a third researcher to decide on the most appropriate coding for that question. This was necessary for 3.5% of the questions.

For each questionnaire or interview, the proportion of questions corresponding to each symptom theme was calculated. Single coded symptom themes were weighted as 1 and dual coded symptom themes were each weighted as 0.5. These counts were then converted into percentages to take into account the overall number of questions in that questionnaire or interview as was done for the symptom categories. These percentages were examined separately for each assessment tool and also averaged across the questionnaires and interviews within a particular disorder (e.g., all the ASD questionnaires and interviews) to provide an aggregate view of each of the 10 disorders, and for the cross-disorder tools (**Figure 3**).

#### Coding and Analysis of Other Question Features

In addition to coding the symptom category and symptom theme, other information embedded within the question content and/or answer options was also coded. This included coding whether the question asked about the presence, duration, severity, timing, or frequency of the symptom. For example, some questions focused on how often a patient experienced a particular symptom while other questions focused on the severity of the symptom experience. In the majority of cases, the answer options within the screening questionnaires provided an indication about the assessment style. For clinical interviews, where questions were often more open-ended, the wording of the question was used to infer the type of information that was being elicited. For example, questions starting with the words “how often” were considered to be assessing the frequency of a symptom, while questions asking about “how much” a symptom bothered someone was considered to be assessing symptom severity. For questions where the wording of the question was more ambiguous, or when a question simply asked whether a person was/had experiencing/experienced a particular symptom with no reference to timing, duration, frequency, or severity, the coding of “presence” was used.

Questions were also coded according to the time period over which the symptom was assessed. For example, some questions asked about symptoms occurring within the past week(s), past month(s), or past year(s) while others asked about symptoms occurring during childhood or across the entire lifetime. For the majority of screening questionnaires, this information was obtained from the introductory portion of the questionnaire. Where this information was not provided on the questionnaire or was more ambiguous, the questions were coded as “no specific time window” unless indicative information was specifically included in the individual questions. For the more open-ended clinical interviews, the time period information was determined based on information provided in the introductory sections of the interview modules, or from the individual question content itself. For example, a question may ask “In the last year…” was coded as “Past Year”, while “Have you ever…” and “did you ever have an episode when…” was coded as “Lifetime”, and “When you were 4/5 years old…” was coded as “Childhood”. Where the time period was more ambiguous in the interview question, or where no time period information was included, it was coded “no specific time window”.

### Symptom Similarity Analysis

To determine the consistency of assessment within a disorder and the overlap between disorders, we computed a symptom similarity score between each pair of questionnaires both within and across disorders. Symptom similarity scores across pairs of assessment tools were calculated for each symptom category as the minimum percentage of questions dedicated to that category divided by the maximum percentage value. We then computed a weighted average using the mean of the two percentage values as the weighting factor to arrive at a questionnaire level similarity score. This meant that symptom level similarity scores were weighted more strongly when the symptom was associated with a greater proportion of questions overall within an assessment tool. We illustrate this with a simulated example with eight symptoms where there is a similar spread of symptoms across the pair of assessment tools (**Table 2**).

**Table 2:** Simulated example of symptom similarity computation.

Symptom	Questionnaire A	Questionnaire B	Symptom Similarity	Weighting Factor	Weighted Similarity
Symptom 1	12%	8%	66.7%	10%	6.67%
Symptom 2	12%	8%	66.7%	10%	6.67%
Symptom 3	12%	10%	83.3%	11%	9.17%
Symptom 4	12%	10%	83.3%	11%	9.17%
Symptom 5	12%	16%	75.0%	14%	10.50%
Symptom 6	12%	16%	75.0%	14%	10.50%
Symptom 7	14%	16%	87.5%	15%	13.13%
Symptom 8	14%	16%	87.5%	15%	13.13%
			Questionnaire Similarity		79%

An overall similarity score was then computed for each disorder by averaging the similarity scores of each pair of assessment tools for that disorder. To compute the symptom overlap *between* disorders we averaged the scores between all pairs of assessment tools, where one of the pair was from each of the disorders (e.g., the average of tools designed to assess PTSD paired with tools designed to assess OCD).

## Results

### Consistency of Assessment Formats

**Figure 1:**
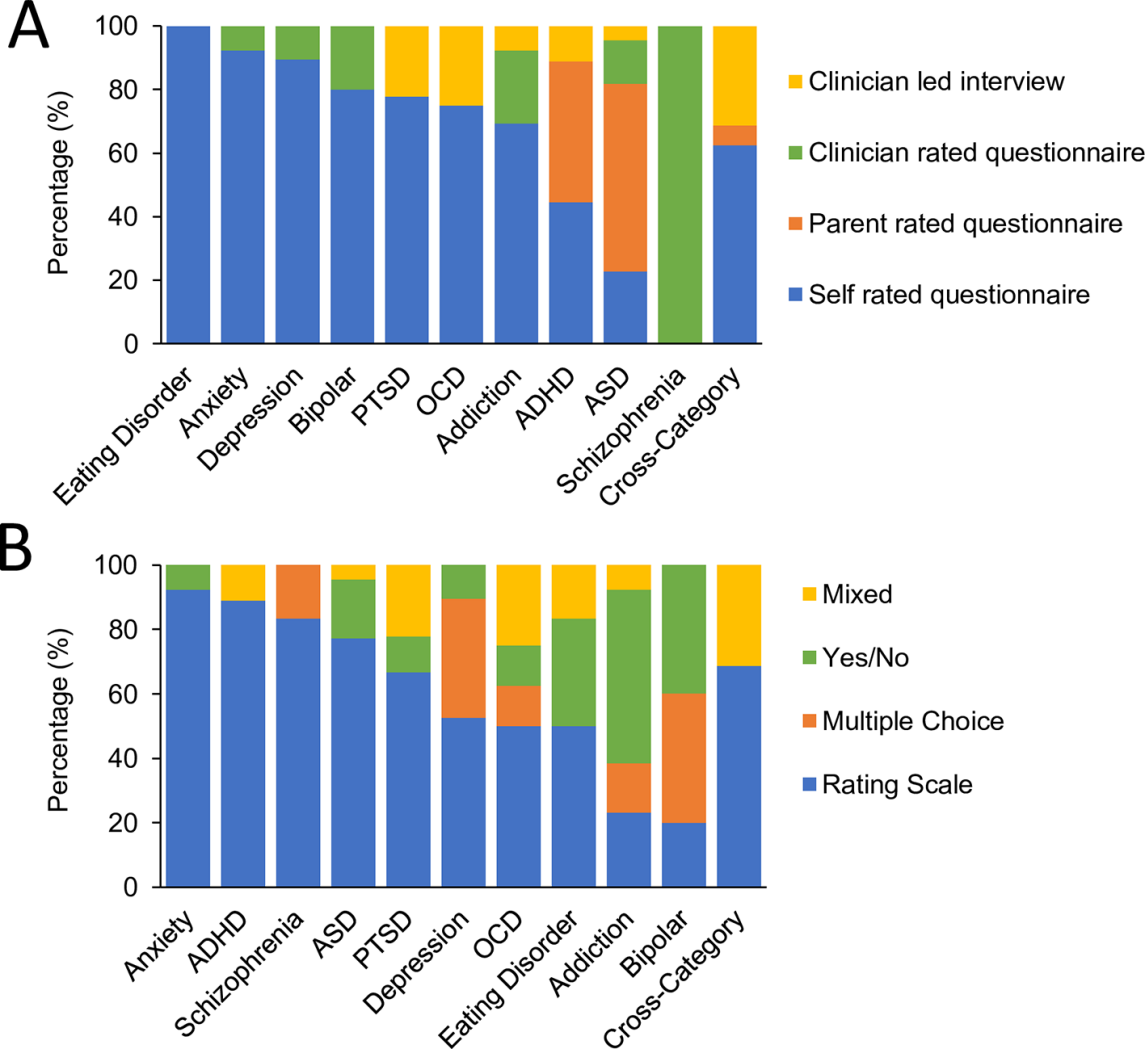
Format of assessment tools. **(A)** Comparative proportion of assessment tools by format of administration; self rated questionnaires (blue), parent rated questionnaires (orange), clinician rated questionnaires (green), or clinician led interviews (yellow). **(B)** Comparative proportion of assessment tools by format of questions; rating scale (blue) a multiple choice list (orange), a yes/no response (green) or a mix of response options (yellow).

Out of the 126 questionnaires and clinical interviews selected for inclusion in the final analysis (**Table 1**, **Supplementary Table 1**) 80 (61 adult) were self-rated, 18 (all pediatric) were parent rated, 16 (14 adult) were clinician rated questionnaires and 12 (7 adult) were clinician led interviews. **Figure 1A** depicts the distribution of assessment types across the different disorders as well as for the cross-category assessment tools. While most disorders were predominantly assessed by self-report, schizophrenia, ADHD, and ASD were predominantly assessed by the parent or clinician based on observation or opinion. This largely reflects the landscape of assessment. However, it also reflects our preferential selection of self-report tools over parent report where both were available for most disorders (e.g., 12 out of 19 self-report pediatric tools (63%) also had parent-rated versions which were not included) and the opposite for ADHD and ASD (1 out of 18 parent rated tools also had an alternative self-rating version which was not included).

In terms of question and answer format, overall 81 (48 adult) assessment tools were answered using rating scales, 20 (15 adult) were answered using a yes/no response option, 12 (11 adult) were answered using a multiple choice and 13 (8 adult) were answered using a mix of different answer formats corresponding to a more open ended style of interview. **Figure 1B** shows the distribution of answer formats across disorders as well as for the cross-category assessment tools. Rating scales dominated the assessment of most disorders while addiction stood out in being largely based on Yes/No answers thereby predominantly assessing presence rather than severity of symptoms. Cross category, OCD, and PTSD were distinct in having a larger fraction of assessments using the mixed answer format associated with a more open-ended interview style.

**Figure 2:**
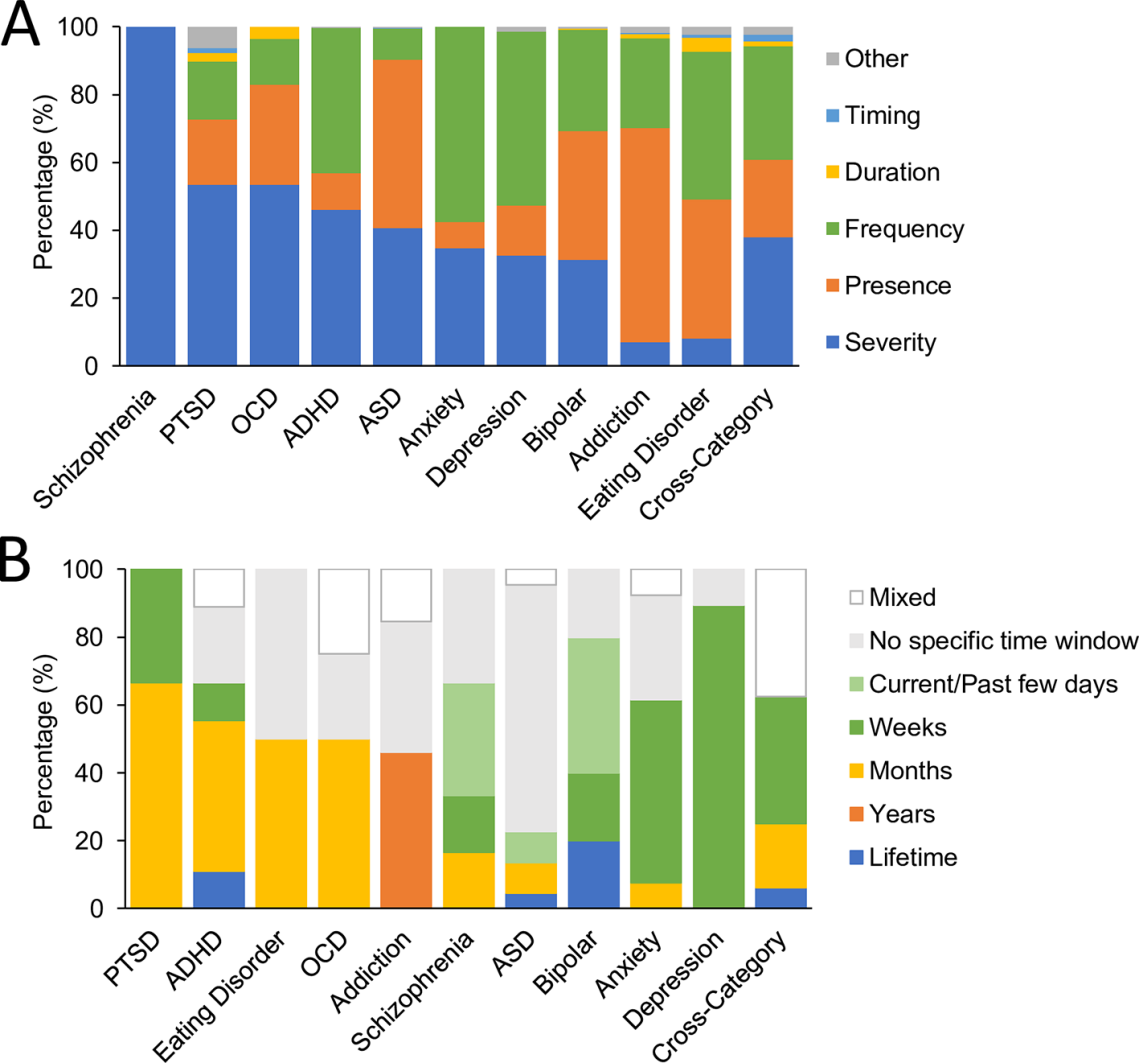
Symptom assessment characteristics. **(A)** Comparative proportion of questions within each disorder and for cross-disorder tools (averaged across assessment tools) by symptom aspects; presence (orange), duration (yellow), severity (dark blue), timing (light blue), or frequency (green) of the symptom. **(B)** Comparative proportion of assessment tools by time period of symptom assessment; currently/past few days (includes past 24 h and past 48 h; pale green), weeks (includes past week and past 2 weeks; dark green), months (includes past month, past 3 months and past 6 months; yellow), years (includes past year and past 2 years; orange), lifetime (includes childhood, adulthood, lifetime and lifetime episodes, blue), no specific time window (gray), and mixed (white).

Examination of other question characteristics revealed considerable variability in terms of whether questions assessed the presence, duration, severity, timing or frequency of the symptom. **Figure 2A** shows the percentage of questions within each assessment tool, averaged across each disorder, which asked about the presence, duration, severity, timing or frequency of the symptom. For schizophrenia, OCD, and PTSD, symptom severity were the predominant assessment formats. In contrast, the frequency of symptoms was the dominant assessment format for depression and anxiety. Assessments of symptom timing or duration were less common across all disorders.

When examining the time period over which symptoms were assessed, a similar pattern of heterogeneity was observed (**Figure 2B**). The most commonly assessed time period of symptoms for ADHD, PTSD, eating disorder, and OCD was the past few months while assessments of bipolar disorder, anxiety and depression more commonly used a time period of the past few days or weeks. In contrast, assessments of ASD did not readily specify a time period for symptoms while addiction assessments either did not specify a time period or used a time period covering the past year(s). There was therefore very little consistency in assessment time period across the different disorders.

### Overview of Symptom Categories, Themes, and Characteristics

**Figure 3:**
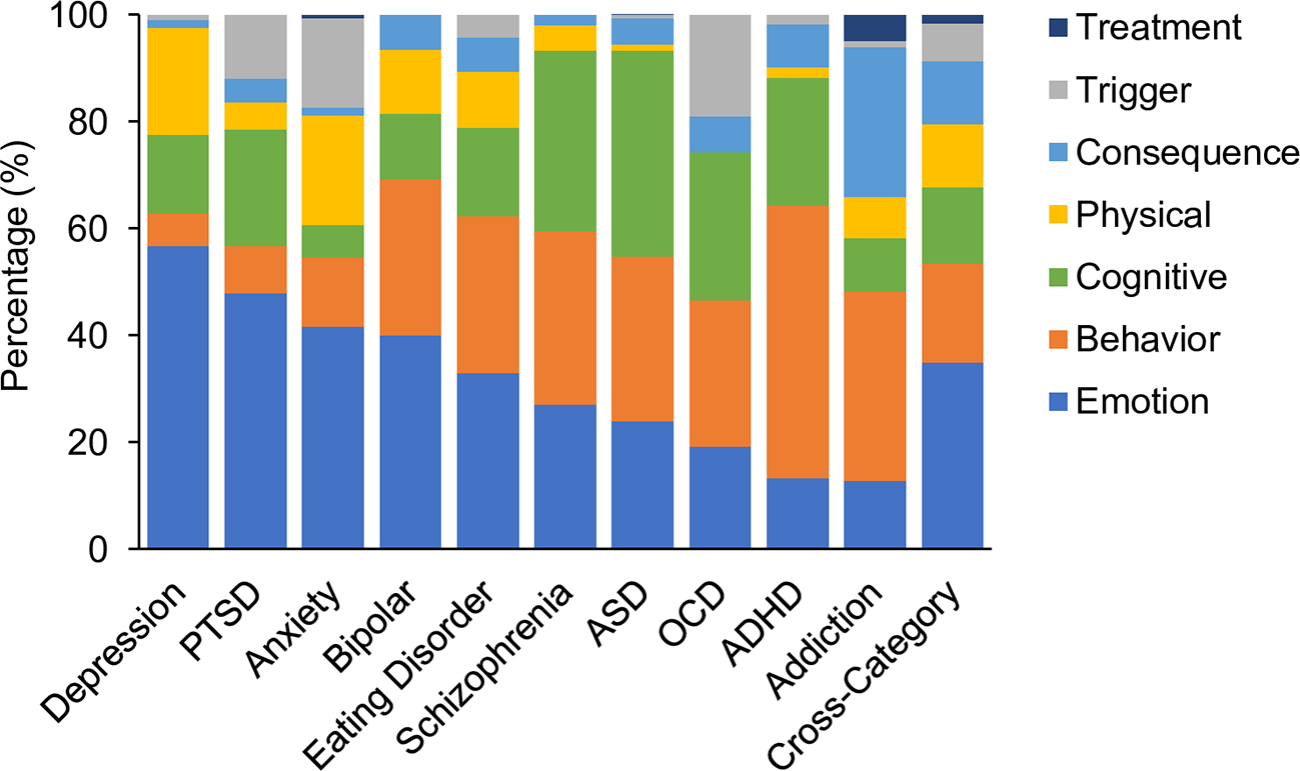
Comparative proportion by symptom theme. Emotion; (mid blue), behavior (orange), cognitive (green), physical (yellow), consequence(s) (pale blue) and trigger(s) (gray), or the treatment(s) of a symptom (dark blue). Values are averages across assessment tools for each disorder and for cross-disorder tools.

Each assessment tool was analyzed along various dimensions to reveal the distribution of symptom themes and symptom categories. **Figure 3** shows the proportion of questions corresponding to each symptom theme averaged across assessments for each disorder. The results showed that in the aggregate, depression alone was dominated by symptoms that related to emotion (57% of questions). The remaining questions were distributed across physical and cognitive symptoms (20% and 15% respectively). In contrast ADHD was dominated by questions relating to behavioral symptoms (51%) while only 13% focused on emotion related symptoms. Others such as OCD, ASD, eating disorder, and schizophrenia were distributed more uniformly across multiple themes. Assessments for OCD, PTSD and anxiety more commonly asked about the triggers associated with a symptom, while assessments of depression, bipolar, ASD, schizophrenia, addiction, and ADHD rarely asked questions of this type. Assessments of addiction and ADHD more commonly asked about the consequences of a symptom, but this was more rarely assessed for other disorders. Symptom treatments were most commonly asked about during assessments of addiction. As might be expected, cross-disorder assessments included questions which covered all symptom themes, with the greatest proportion of questions asking about emotions.

**Figure 4:**
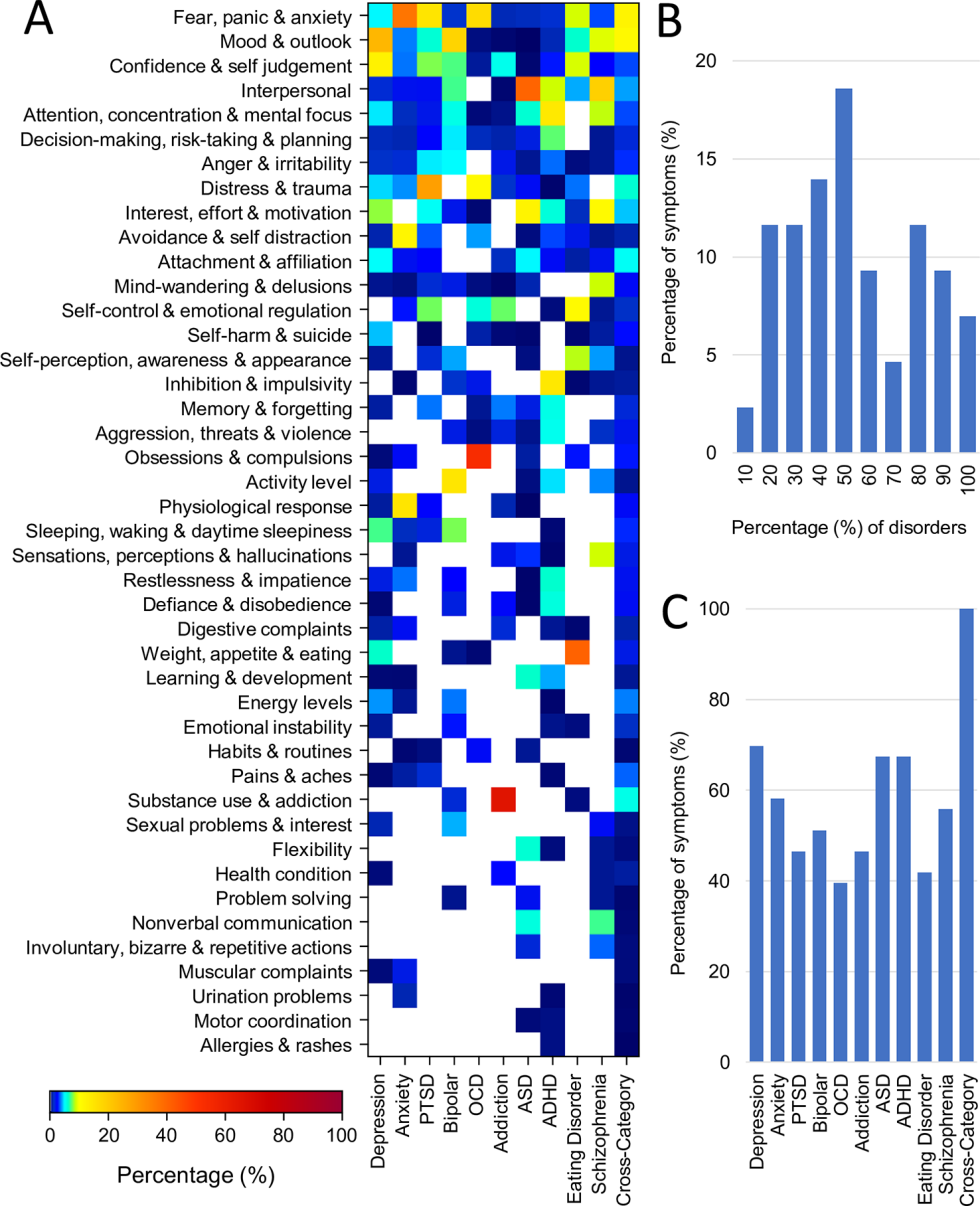
Representation of symptom categories across disorders. **(A)** Proportion (%) of questions from each of the 43 symptom categories for each disorder (averaged across assessment tools) and for cross disorder tools. **(B)** Symptom overlap across disorders. First and last bars represent fraction of symptoms belonging to only one disorder and 100% of disorders respectively. **(C)** Breadth of symptoms assessed for each disorder and for cross-disorder tools. All but one disorder encompassed more than 40% of all symptoms.

**Figure 4A** shows the percentage representation of each of the 43 symptom categories across each of the disorders as well as for the cross-disorder questionnaires (also see **Supplementary Table 3**). The primary takeaway is the large degree of symptom overlap across disorders. However, a number of symptom categories were dominant in only one disorder. For example, “Substance use and Addiction” was dominant for addiction (65% of addiction assessment) as well as being a symptom assessed in bipolar disorder. In contrast, other symptom categories such as “Fear, Panic & Anxiety”, “Mood & Outlook”, “Confidence & Self-Judgement”, “Interpersonal”, and “Attention & Concentration & Mental Focus” dominated assessments of multiple disorders. Fear, Panic, & Anxiety for instance was highest for anxiety (37%) but was also substantially represented in the related disorders of PTSD (15%) and OCD (17%). The percentage of disorders represented by different symptom categories are shown in **Figure 4B** (excluding cross-category assessment tools). Overall three symptom categories (7%) were represented across all disorders and 4 (9%) were represented in all but one disorder. In the aggregate, 42% of symptom categories were represented in over half of all disorders. There was only one symptom category that was assessed by only one disorder (Allergies & Rashes in ADHD assessment), representing a physical symptom that may or may not be specifically relevant to a mental health disorder.

Conversely, all disorders encompassed multiple symptom categories. The distribution is shown in **Figure 4C**. OCD was the most symptom specific covering only 39% of symptom categories followed by eating disorder (42%), addiction and PTSD (both 47%). In contrast depression was the most general, encompassing 70% of all symptom categories followed by ASD and ADHD (both 67%). While the weighting of these overlapping symptoms certainly differs across disorders, it nonetheless demonstrates that no individual symptom category can be considered a definitive element of any disorder categorization.

### Similarity Scores Within and Across Disorders

**Figure 5:**
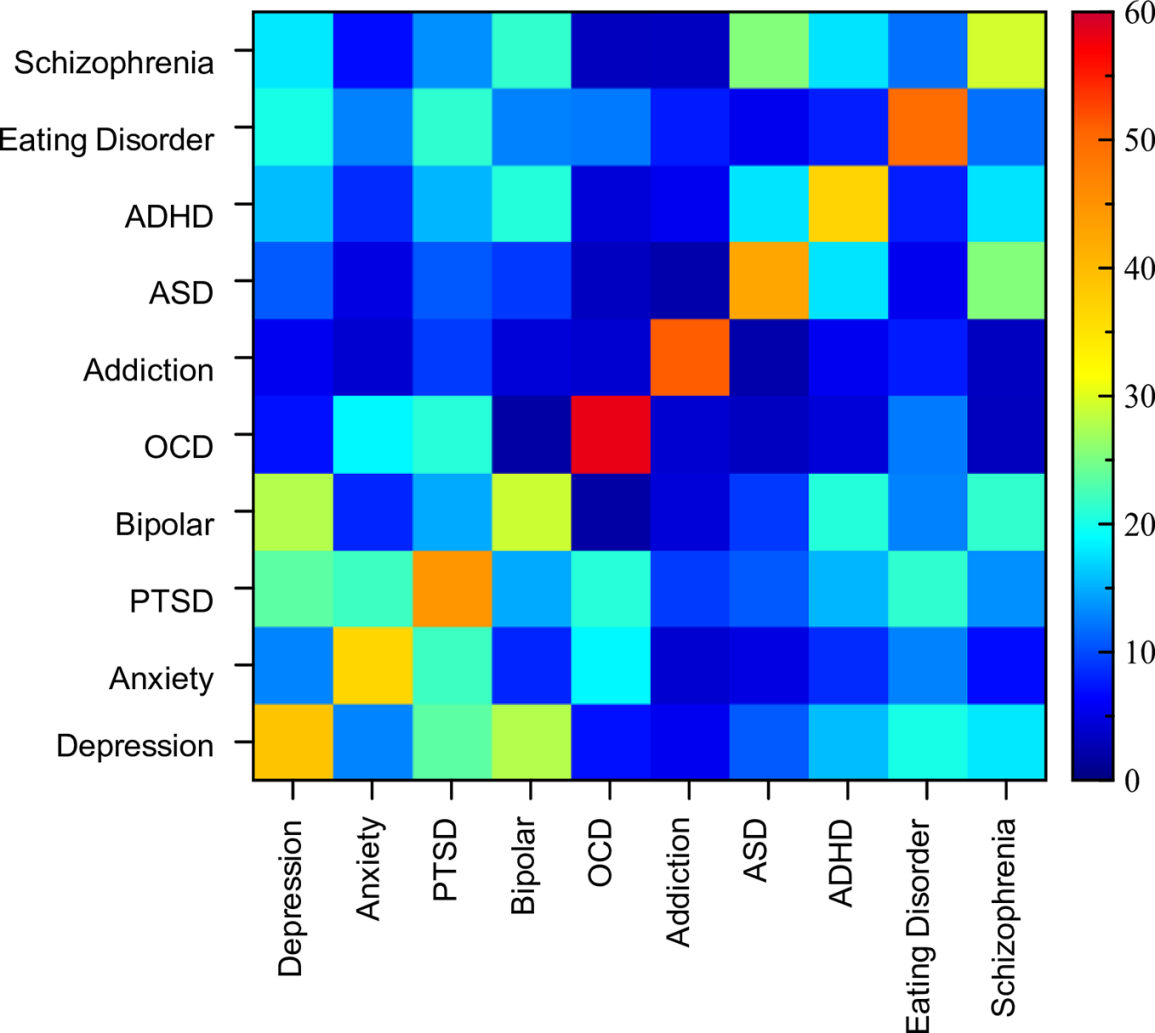
Matrix of similarity scores. Symmetric matrix of similarity scores between assessment tools both within and between disorders. Diagonal indicates within disorder similarity comparisons and ranged from 29% (bipolar) to 58% (OCD).

The previous sections illustrate the large heterogeneity in assessment format as well as the substantial overlap in the range of symptoms across disorders. However, the particular weighting of symptoms in the assessments varied (i.e., the number of questions dedicated to a particular symptom category). Therefore, to get a better sense of the distinctions between disorders along this dimension, we computed a measure of symptom consistency across disorders (see Materials and Methods). **Figure 5** shows a (symmetric) matrix of the average similarity scores within and between disorders (values pertaining to this figure can be found in **Supplementary Table 3**).

Analysis of similarity scores between pairs of disorders ranged from 2% to 28%. The most similar assessments (i.e., above 20%) from a cross-disorder perspective were between PTSD and OCD, depression and PTSD, anxiety and PTSD, PTSD and eating disorder, ASD and schizophrenia, depression and bipolar disorder, and bipolar disorder and schizophrenia. The disorder pairs that had the lowest similarity scores (under 4%), and therefore were most dissimilar in terms of symptoms considered, were OCD and ASD, OCD and bipolar disorder, OCD and schizophrenia, ASD and addiction, and schizophrenia and addiction. Thus, while the range of symptoms overlapped considerably across many disorders, the distinctions between them were largely at the level of importance ascribed to the symptom.

While on the face of it, this appears to support the specific divisions of disorders, the disorder comparison analysis was done based on an average of the tools within any individual disorder. However, when looking at the overall consistency of assessment of any individual “disorder”, symptom similarity was surprisingly low (diagonal of the matrix in **Figure 5**). OCD showed the greatest level of assessment consistency (58% similarity across 8 tools), followed by addiction (51% across 13 tools). The other disorders showed much lower consistency with assessments of bipolar disorder and schizophrenia being the lowest at 29%. Thus, when there is substantial symptom ambiguity within disorders, it calls into question the relevance of comparing between two disorders (as we have done above) as individual assessment tools can have vastly diverging similarity scores. For example, the overall similarity between depression and ADHD was 16%, but when looking at individual pairs, PHQ-9 (depression), and CAARS (ADHD) were 37% similar, while PHQ-9 (depression) and ASRS-5 (ADHD) were only 8% similar.

### Individual Disorders

We next present the consistency across individual assessment tools within each disorder separately. The consistency of symptom themes and categories among individual assessments, as well as the similarity scores, are reported. For completeness, we also report the breadth of symptoms, with the caveat that this metric is influenced by the total number of questions within an assessment tool, especially when the number of questions were lower overall. For example, an assessment tool with only 15 questions cannot cover a wide breadth of symptoms, while an assessment tool with 700 questions could potentially do so.

#### Depression

**Figure 6:**
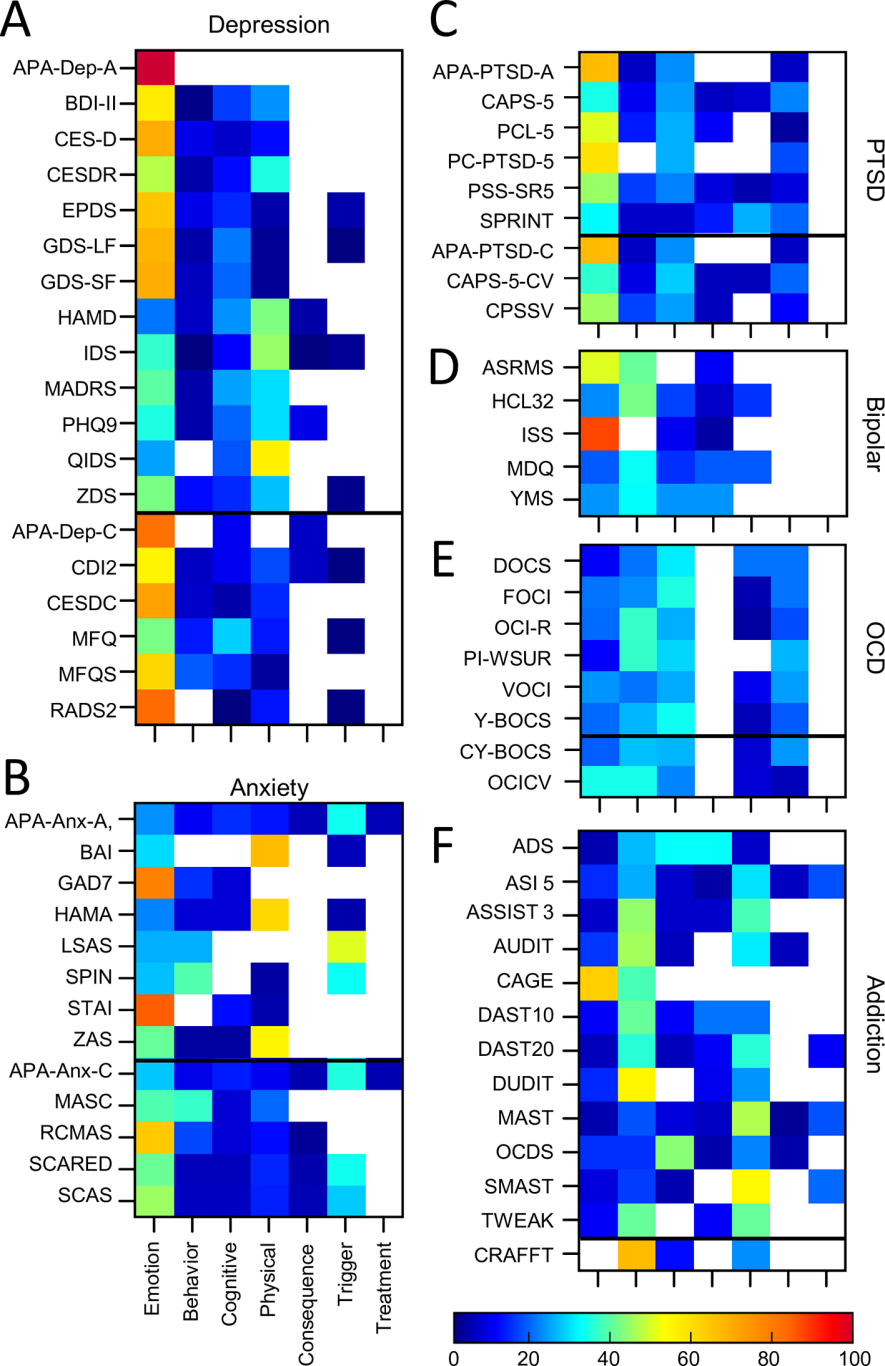
Proportion of symptom themes. Proportion of symptom themes (%) across individual assessment tools within individual disorders. **(A)** Depression. **(B)** Anxiety. **(C)** PTSD. **(D)** Bipolar. **(E)** OCD. **(F)** Addiction. Black line denotes delineation between adult (top) and pediatric (bottom) tools. See **Supplementary Table 5** for a list of abbreviations.

Nineteen auxiliary depression assessment tools were analyzed. Thirteen of these were predominantly developed for adult populations and 6 for pediatric populations (see **Table 1**, **Supplementary Table 1**). Two other common assessments of depression (the DASS: Depression, Anxiety Stress Scales (137, 138); and HADS: Hospital Anxiety and Depression Scale; (139) were excluded from this study as they spanned two disorders of interest. **Figure 6A** shows the considerable variability of depression assessments in terms of symptom themes (see also **Supplementary Table 3**). For example, questionnaires such as HAMD, QIDS, and IDS included a lower proportion of emotion themed questions (ranging from 21% to 37%) and a higher proportion of physical themed questions (ranging from 43% to 56%), compared to the other assessment tools. In contrast, the APA-Dep-C, APA-Dep-A, RADS2, CESDC, CES-D, and GDS-SF had a greater proportion of emotion themed questions (ranging from 70% to 100%). None of the questionnaires prioritized cognitive or behavioral symptoms within their questions (a maximum of 29% for cognitive symptoms and a maximum of 19% for behavioral symptoms across all depression assessments). In addition, none of the depression assessments asked about previous or current treatments, and only 37% and 26% of assessments asked about symptom triggers and consequences respectively.

Symptom categories also varied considerably across assessments. Seventy percent of all symptom categories were represented across all the assessment tools together. However, only 10% of these were common across 80% or more assessment tools (Confidence & Self judgement; Mood & Outlook; Interest, Effort & Motivation). The similarity analysis showed that the depression assessments were, on average, only 39% similar. One of the tools (HAMD) included 4 questions which were not used in the scoring for depression. When HAMD was removed from the analysis, the average similarity score moved up by one percentage point to 40%. Similarity scores between individual pairs of assessments ranged from 7% (APA-Dep-A and QIDS) to 94% (CES-D and CES-DC). Comparisons between adult and pediatric tools showed that adult tools were, on average, 40% similar, pediatric tools were 46% similar, and the similarity score between adult and pediatric tools was 36%, suggesting there were some differences between the two groups. See **Supplementary Table 4** for a table of similarity scores between each depression assessment tool.

#### Anxiety Assessments

Thirteen auxiliary anxiety assessment tools were included in the analysis of which 8 were for adult populations and 5 were pediatric (see **Table 1** and **Supplementary Table 1** for details). One additional common anxiety assessment (RCADS, Revised Children's Anxiety and Depression Scale) was excluded from this study due to its cross-disorder approach. We note that two assessment tools (APA-Anx-A and APA-Anx-C) were composed of a set of related APA assessments each focusing on different types of anxiety (covering generalized anxiety, panic, agoraphobia, social phobia, separation anxiety and social anxiety which we have aggregated together as one broad assessment of anxiety. In addition, 2 assessment tools (LSAS and SPIN) included a greater number of questions on social anxiety. We performed the analysis both with and without these two assessment tools to determine whether including them skewed the results in any way but found only one minor change in the results (see below) so we included them in the analysis.

Looking across all the anxiety tools, emotion themed symptoms dominated in GAD7 and STAI (79% and 85% respectively) while BAI, HAMA, and ZAS emphasized physical symptoms (ranging from 55% to 67% of questions; **Figure 6B**, **Supplementary Table 3**). Many of the assessment tools (62%) also asked about symptom triggers. In general, the pediatric tools were more comprehensive in terms of covering all symptom themes (with the exception of treatment) compared to the adult tools. For example, all but one of the pediatric tools (but only 1 of the adult tools) asked about consequences of a patient’s symptoms. In addition, all pediatric tools included questions about cognitive and behavioural symptoms, while there were gaps and heterogeneity across the adult assessment tools.

Fifty-eight percent of symptoms were covered across all anxiety assessment tools. However, only 1 symptom (Fear, Panic, & Anxiety) was assessed by 80% or more of the assessment tools. This resulted in, on average, only a 37% similarity across all assessment tools. One reason for this relatively low similarity score could be due to the fact that anxiety has multiple dimensions (e.g. phobia, separation anxiety, social anxiety) which could be differentially assessed across tools. In addition, some assessments asked about specific symptoms occurring over the past few weeks (GAD7), whilst others (especially for pediatric tools) were more trait-like and asked about the degree to which an individual generally experienced symptoms associated with anxiety (e.g., RCMAS), which could also have impacted the overall similarity score. Similarity scores between individual pairs of assessments ranged from 6% (LSAS and ZAS) to 86% (APA-Anx-C and APA-Anx-A; See **Supplementary Table 4** for details). One of the tools (SCAS) included 6 questions which were not used in the scoring for anxiety. However, when SCAS was removed from the analysis, the average similarity score did not change. To explore the impact of including LSAS and SPIN we examined the average similarity scores between these two assessment tools and all the other assessment tools and showed that they were, on average, 36% similar to the other tools, which was not very different from the average overall similarity score of 37% above. Comparisons between adult and pediatric tools showed that adult tools were, on average, 31% similar, pediatric tools were 53% similar, and the similarity score between adult and pediatric tools was 37%, suggesting that pediatric tools were considerably more similar than adult tools.

#### PTSD Assessments

Nine PTSD assessment tools were included; six assessments for adult populations, and three assessments for pediatric populations (see **Supplementary Table 1** for details). Two tools were diagnostic (clinician led) interviews, whilst the remaining were auxiliary (self report). Emotion themed symptoms dominated most PTSD assessment tools (ranging from 31% to 67%; **Figure 6C**, **Supplementary Table 3**). Furthermore, with the exception of PC-PTSD-5, all assessment tools also included cognitive, behavioral and trigger themed symptoms. Six out of the nine assessment tools also included physical themed symptoms and four assessments considered consequences. None asked about symptom treatment.

Forty-seven percent of symptoms were asked about in total across all PTSD assessments of which only 15% were common across 80% or more assessment tools (Confidence & Self judgement; Fear, Panic & Anxiety; Distress & Trauma). This resulted in a 45% similarity score on average across all PTSD assessments. Similarity scores between individual pairs of assessments ranged from 16% (PCL-5 and SPRINT) to 100% (APA-PTSD-A and APA-PTSD-C; See **Supplementary Table 4** for details). We also examined the impact of including both diagnostic and auxiliary tools on the similarity scores and showed that the diagnostic interviews were 45% similar to the auxiliary tools, while the similarity scores of the auxiliary tools alone was 43%. Comparisons between adult and pediatric tools showed that adult tools were, on average, 40% similar, the 3 pediatric tools were 44% similar, and adult and pediatric tools were 48% similar, influenced by the fact that two pediatric tools had corresponding adult versions.

#### Bipolar/Mania Assessments

Five bipolar/mania assessment tools were included in the analysis, all adult (See **Supplementary Table 1** for details). Of these, ISS and ASRMS covered a greater proportion of emotion themed symptoms compared to the other assessment tools (88% and 50% respectively; **Figure 6D**, **Supplementary Table 3**). The scales varied in terms of the proportion of questions that asked about behavioural symptoms (ranged from 0% to 41%), cognitive symptoms (ranged from 0% to 23%) and physical symptoms (ranged from 3% to 23%). None of the assessment tools asked about symptom treatments or triggers, while two out of the five assessment tools asked about consequences of the symptoms.

Fifty-one percent of symptoms were asked about in total, of which only 23% of symptoms were assessed by 80% or more of the assessment tools (Activity Level; Sleeping, Waking & Daytime Sleepiness; Confidence & Self judgement; Mood & Outlook; Anger). On average, bipolar assessments were 29% similar, among the lowest consistency of any disorder covered here. Here again, one tool (HCL32), used only 32 questions (out of a possible 43) in the actual scoring. When HCL32 was removed from the analysis then the average similarity score decreased to 26%. Similarity scores between individual pairs of assessments ranged from 16% (ISS and YMS) to 41% (ISS and HCL32; see **Supplementary Table 4** for details).

#### OCD Assessments

Eight OCD assessments were analyzed. This included 6 for adult populations and two for pediatric populations (See **Supplementary Table 1** for details). **Figure 6E** shows the distribution of symptom themes across each OCD assessment tool showing a roughly even proportion of questions across multiple themes and a general consistency in their proportions across the assessments (see also **Supplementary Table 3**). All the OCD assessment tools contained questions themed as emotion (ranged from 10% to 33%), cognitive (ranging from 21% to 34%) and behavioral (ranged from 20% to 36%) symptoms. All assessment tools also asked about symptom triggers (ranged from 5% to 26% of questions) and all but one asked about consequences (ranged from 3% to 20% of questions). None of the assessment tools included in this analysis asked about physical symptoms or treatment.

The symptom categories across all OCD assessment tools were relatively compact encompassing only 40% of all symptoms. 12% of these were found in 80% or more of the assessment tools (Obsessions & Compulsions; Distress & Trauma). OCD assessment also had the highest similarity on average of all disorders (58%). For both YBOCS™ and CYBOCS™, only the scale element (excluding 2 questions) and not the checklist element of the tool contributed to the actual scoring algorithm. When these assessments were excluded from the analysis, the average similarity score decreased to 55% and the two clinician led assessment tools were, on average, 59% similar to the other PTSD assessment tools. Similarity scores between individual pairs of assessments ranged from 41% (PI-WSUR and OCI-R) to 90% (Y-BOCS™ and CY-BOCS™; see **Supplementary Table 4** for details). Comparisons between adult and pediatric groups showed that adult tools were, on average, 55% similar, the two pediatric tools were 54% similar, and adult and pediatric tools were 63% similar, influenced by the fact that the two pediatric tools had corresponding adult versions.

#### Addiction Assessments

Thirteen addiction assessment tools were included of which 12 were developed primarily for adult populations and one for pediatric (adolescent) populations (see **Supplementary Table 1** for details). Seven of these focused on alcohol addiction (ADS, AUDIT, CAGE, MAST, OCDS, SMAST, TWEAK), 3 focused on drug addiction (DUDIT, DAST10, DAST20), and 3 focused on both alcohol and drug addiction (CRAFFT, ASI5, ASSIST3). Assessments pertaining to other forms of addiction (e.g., gambling, gaming) were not included so as to limit the scope to the most common forms of clinical addiction.

There was considerable heterogeneity in symptom themes when looking across all addiction assessments (**Figure 6F**, **Supplementary Table 3**). For example, two tools included questions which covered all seven themes (the MAST and ASI-5) while other assessment tools only covered two or three themes (e.g., CAGE, CRAFFT). The majority of the addiction assessments included here focused primarily on behavioral symptoms (ranging from 14% of questions for OCDS through to 67% of questions for CRAFFT). However, a number of assessment tools also focused strongly on emotional symptoms (e.g., CAGE, 63% of questions), cognitive symptoms (e.g., OCDS, 43% of questions) or physical symptoms (e.g., ADS, 32% of questions). All but one tool asked questions on symptom consequences, and a number also included questions which asked about symptom triggers and treatment.

When examining the breadth of symptoms assessed across all addiction assessment tools, the results show that 47% of all symptoms were covered across all assessments of which only 1 symptom was common across 80% or more assessment tools (Substance Use & Addiction). However, despite this, on average the addiction assessment tools included here were 51% similar. Similarity scores between individual pairs of assessments ranged from 19% (TWEAK and ASI-5) to 89% (ASSIST3 and SMAST; See **Supplementary Table 4** for details). When assessing alcohol addiction and drug addiction separately, the average similarity scores was 51% between alcohol addiction assessments, 57% between drug addiction assessments and 38% between tools assessing both drug and alcohol addiction. The ASI clinical interview which assessed both drug and alcohol addiction included a section at the end on general psychiatric status and showed the greatest dissimilarity from the other addiction assessments (it was on average only 25% similar to all the other addiction assessment tools). When the ASI was excluded from the analysis the overall average similarity score went up from 51% to 55%.

#### ASD Assessments

**Figure 7:**
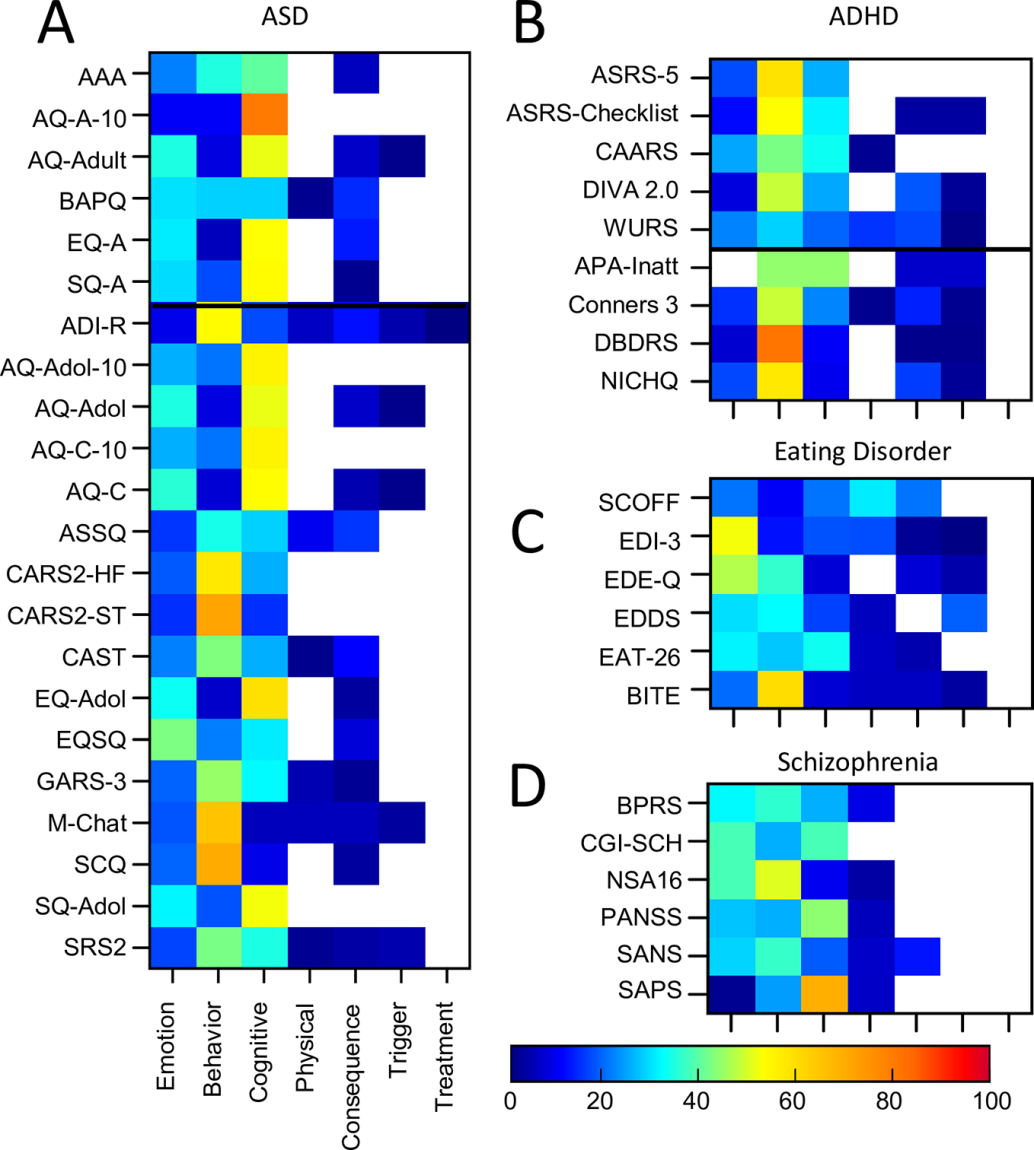
Proportion of symptom themes. Proportion of symptom themes (%) across individual assessment tools within individual disorders (continued). **(A)** ASD. **(B)** ADHD. **(C)** Eating Disorder. **(D)** Schizophrenia. Black line denotes delineation between adult (top) and pediatric (bottom) tools. See **Supplementary Table 5** for a list of abbreviations.

Twenty-two ASD assessment tools were included of which 6 were primarily developed for adult populations and 16 assessment tools for pediatric populations (see **Supplementary Table 1** for details). All assessments included emotion, cognitive and behavioural themed symptoms (**Figure 7A**, **Supplementary Table 3**). However, there was considerable heterogeneity in terms of which theme was dominant in any one assessment tool. For example, approximately half of the assessment tools comprised of questions where the assessment of behavioral symptoms was dominant. In contrast, the other ~half of assessment tools comprised of questions where the assessment of cognitive symptoms was dominant (and this included all adult tools). Across all ASD assessment tools, the proportion of questionnaires that focused on cognitive symptoms ranged from 5% (M-CHAT) to 80% (AQ-A-10), while the proportion of questionnaires which focused on behavioral symptoms ranged from 5% (EQ-A) to 71% (CARS2-ST) indicating the high level of variability across the different assessment tools. In addition, only one tool, the EQSQ, predominantly focused on emotional symptoms (42%).

Sixty-seven percent of symptoms were evaluated in total across all ASD assessments. Of these, only 10% of symptoms were common across 80% or more assessment tools (Attachment & Affiliation; Interpersonal; Interest, Effort & Motivation). This resulted in a similarity score of only 43% overall, demonstrating a fairly high level of inconsistency. One questionnaire (CAST) included seven questions (out of 38) which were not used for the scoring. When CAST was excluded from the analysis, the average similarity score shifted by one percentage point to 42%. Similarity scores between individual pairs of assessments ranged from 3% (M-CHAT and SQ-A) to 100% (AQ-Adol and AQ-C; AQ-Adol and AQ-A; AQ-A and AQ-C; EQ-A and EQ-Adol; see **Supplementary Table 4** for details). The average similarity between the diagnostic assessment tool (ADI-R) and all the other auxiliary tools was 41%, similar to the overall similarity score of 43%. In addition, as the adult ASD tools were predominantly self-rated and the pediatric tools were predominantly parent-rated we compared these two groups separately. The average similarity scores were as follows: 39% within the adult tools; 44% within the pediatric tools; and 42% between the adult and pediatric tools.

#### ADHD Assessments

Nine ADHD assessment tools (eight auxiliary) were included of which five were for adult populations and four were for pediatric populations (see **Supplementary Table 1** for details). Three of these pediatric tools (Conners3™, DBDRS, and NICHQ) were also designed to assess symptoms relating to conduct disorder and oppositional defiance disorder.

The majority of the ADHD assessment tools focused on behavioral symptoms (ranging from 28% to 81% of questions; **Figure 7B**, **Supplementary Table 3**). This was most apparent for DBDRS, ASRS-5, and NICHQ (with 81%, 58%, and 57% of questions, respectively). Questions assessing cognitive symptoms were also common in the majority of assessment tools (ranging from 9% for NICHQ through to 44% APA-Inatt). The inclusion of questions which assessed emotional symptoms was relatively low (ranging from 0% for APA-Inatt to 24% for CAARS) and only three out of the nine tools asked about physical symptoms. All tools but two (ASRS-5, CAARS) asked about both triggers and consequences of the symptoms although none asked about symptom treatment.

Across all ADHD assessment tools 67% of symptoms were asked about in total of which 21% of symptoms were assessed by 80% or more of the assessment tools (Attention & Concentration & Mental Focus; Decision-making, Risk taking & Planning; Restlessness & Impatience; Inhibition & Impulsivity; Interpersonal; Interest, Effort & Motivation). However, there was only 37% similarity overall across ADHD assessment tools. Similarity scores between individual pairs of assessments ranged from 11% (ASRS-Checklist and WURS) to 68% (ASRS-Checklist and DIVA 2; See **Supplementary Table 4** for details). In addition, one questionnaire (WURS) used only 25 (out of 61) questions in the scoring algorithm for ADHD while another (ASRS-Checklist) only used the first 6 questions (out of 18) for scoring. Exclusion of the WURS from the analysis increased the overall average similarity score to 40%, while exclusion of the ASRS-Checklist resulted in no change. The average similarity score across the auxiliary tools only (i.e., without DIVA 2) was 35%. Furthermore, to determine the impact of including assessment tools that considered symptoms for conduct disorder (CD) and oppositional defiance disorder (ODD), we examined the similarity scores for these two groups separately. The average similarity score between the +CD/ODD and -CD/ODD groups of assessment tools was 32%, while the average similarity within the -CD/ODD group (6 assessment tools) was 38% and the average similarity within the +CD/ODD group (three assessment tools) was 63%. In addition, as the adult ADHD tools were predominantly self-rated (except DIVA-2) and the pediatric tools were predominantly parent-rated we compared these two groups separately. The average similarity scores were 40% within the adult tools, 42% within the pediatric tools and 34% between the adult and pediatric tools indicating that there may be some differences between adult and pediatric tools.

#### Eating Disorder Assessments

Six eating disorder assessment tools were included (all adult/adolescent; all auxiliary; see **Supplementary Table 1** for details). The majority of the tools focused on behavioral (ranging from 10% to 60% of questions) and emotional symptoms (ranging from 19% to 52% of questions; **Figure 7C**, **Supplementary Table 3**). A number of tools (EAT26, EDI-3, EDDS) also focused on cognitive symptoms (33%, 17%, 16% respectively). With the exception of EDI (17%) and SCOFF (30%), the inclusion of questions which assessed physical symptoms was typically lower. All but 2 tools (SCOFF and EAT26) asked about triggers and all but 1 (EDDS) asked about consequences while no tools asked about symptom treatment.

Across all eating disorder assessment tools 42% of symptoms were asked about in total of which 17% of symptoms were assessed by 80% or more of the assessment tools (Fear, Panic & Anxiety; Confidence & Self Judgement; Self-perception, Awareness & Appearance; Weight, Appetite, & Eating; Self Control & Emotional Regulation). There was 50% similarity overall across eating disorder assessment tools and similarity scores between individual pairs of assessments ranged from 30% (EAT-26 and SCOFF) to 79% (BITE and EDDS; See **Supplementary Table 4** for details).

#### Schizophrenia Assessments

Six clinician rated schizophrenia assessment tools for adult populations were included (all auxiliary; See **Supplementary Table 1** for details). **Figure 7D** shows there was considerable variation across the themes dominating these tools as follows: emotional (1% to 38%), cognitive (9% to 69%), behavioral (24% to 50%), and physical (0% to 8%; see also **Supplementary Table 3**). There was rarely a focus on the consequences, triggers, or treatment of the symptoms.

Fifty-six percent of symptoms were covered across all assessments of which only 13% of symptoms were common to 80% or more of the assessment tools (Attention & Concentration & Mental Focus; Interpersonal; Self-perception, Awareness & Appearance). The 6 assessment tools were, on average, only 29% similar. The differential focus on positive vs negative symptoms of schizophrenia across assessments in part contributed to the dissimilarity across assessments. Similarity scores between individual pairs of assessments ranged from 8% (CGI-SCH and PANSS) to 52% (BPRS and PANSS; See **Supplementary Table 4** for details).

### Cross Disorder Assessments

In addition to the assessment tools which were developed around the 10 disorder types, 16 cross-disorder tools were also included. These included nine assessments for adult populations and seven for pediatric populations (See **Supplementary Table 1** for details). Five of these tools were diagnostic and the remaining were auxiliary. For the modular clinical interviews and questionnaires (CIDI CAPI, DISC-IV, SCID-5, MINI, KSADS, PROMIS-QB-A, PROMIS-QB-C) only those modules which were related to the 10 disorders of interest were included.

Cross-disorder assessments, if comprehensive, should cover all symptoms and themes across these 10 disorders. Indeed 100% of symptoms were asked about across all tools in total (**Figure 4**). However, only 21% of symptoms were assessed by 80% or more of the assessment tools (Attention & Concentration & Mental Focus; Energy Levels; Pains & Aches; Attachment & Affiliation; Confidence & Self Judgement; Anger & Irritability; Fear, Panic & Anxiety; Mood & Outlook; Interest, Effort & Motivation). The maximum symptom categories covered by any single cross-disorder tool was 86% (SCID-5-CV) indicating that none of the available tools covered the full breadth of mental health symptoms corresponding to these 10 disorders.

**Figure 8:**
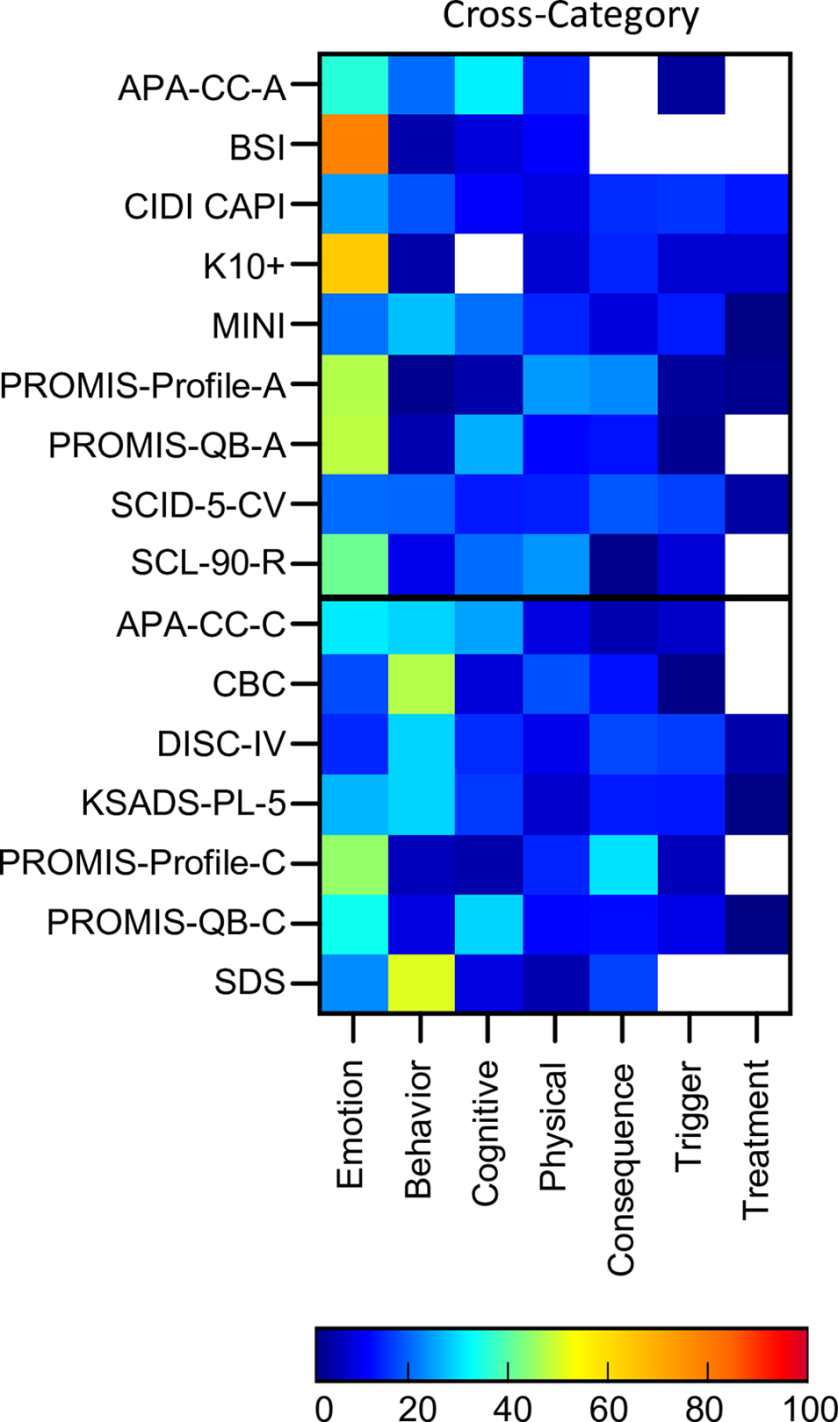
Proportion of symptom themes. Proportion of symptom themes (%) across cross disorder assessment tools. Black line denotes delineation between adult (top) and pediatric (bottom) tools. See **Supplementary Table 5** for a list of abbreviations.

In terms of symptom themes, many cross-disorder tools were biased in terms of symptom theme (**Figure 8**, **Supplementary Table 3**). For instance, the BSI and K10+ were primarily emotion focused (78% and 63% respectively) while only 14% of questions were emotion themed in others (DISC-IV). Others such as the CBC and SDS were more heavily weighted towards behavioural symptoms (47% and 50% respectively). The majority of tools asked about symptom triggers and consequences, while only half asked about symptom treatment.

On average, the cross-disorder tools were only 32% similar with similarity scores for individual pairs of assessments ranging from 9% (BSI and K10+) to 65% (KSADS and DISC-IV; see **Supplementary Table 4** for details). To determine whether there was any difference between diagnostic and auxiliary tools we computed similarity scores for each group separately as well between them. This showed that, on average, auxiliary tools were 29% similar, diagnostic tools were 58% similar, and diagnostic and auxiliary tools were 31% similar. Thus, the diagnostic interviews were considerably more alike compared to the auxiliary tools. Comparisons between adult and pediatric tools showed that adult tools were, on average, 32% similar, pediatric tools were 32% similar, and the similarity score between the adult and pediatric tools was 33%, suggesting that the groups were comparable in terms of the similarly between tools.

## Discussion

Mental health determination is reliant, first and foremost, on the way that a patient’s symptoms are assessed. The questions included on a questionnaire, or asked during an interview, determine the responses which are used within clinical and research domains to reveal psychological challenges, identify neurobiological disruptions or understand socio-environmental impacts. Here our analysis of 110 assessment tools spanning 10 different disorders, as well as 16 which took a cross-disorder perspective, revealed the high level of inconsistency in this expansive assessment landscape which, in turn, has consequences for research and clinical reproducibility, as well as for the wider system of mental health diagnosis and evaluation. This inconsistency was apparent when comparing assessment tools across different disorders, as well as when comparing those intended to assess the same disorder. Significantly, the variability extended not just to how symptoms were being assessed, but to which symptoms were assessed.

### An Inconsistent Approach to Mental Health Assessment

Our analysis of the similarity of symptoms between assessments of the same disorder revealed similarity scores ranging from 29% for bipolar disorder and schizophrenia to 58% for OCD. Between individual pairs of questionnaires assessing the same disorder, there were many cases where similarity of symptoms was lower than 20% (e.g., PCL-5 and SPRINT for PTSD; STAI and ZAS for anxiety, BSI and K10+ for cross-category). Thus, tools designed to assess the same disorder(s) showed considerable inconsistency in the symptoms that they were considering. Consequently, two experimental studies assessing patients with the same clinical diagnosis, but using different tools to assess symptom severity, may deliver different results because they are assessing a different set of symptoms. Furthermore, when combined with differences in the manner a symptom is assessed and the comorbid nature of symptom experience across individuals (140–142), the difficulties in searching for consistency and reproducibility are amplified.

While on one hand there were inconsistencies in symptom assessment within disorders, on the other hand there was also considerable overlap between disorders. Sixty percent of all mental health symptoms were included in the assessment of at least half of all disorders. Furthermore, 7% of all symptoms were assessed within all disorders, while a further 9% were assessed within all but one disorder. Similarity scores between disorders, which considered the differential weighting of symptoms between disorders, could be as high as 28% overall (depression and bipolar disorder) and 59% between two individual questionnaires (SANS, schizophrenia and SCQ, ASD, which similarly assess symptoms of Attachment & Affiliation; Interpersonal; Nonverbal Communication; Self-perception, Awareness & Appearance; Interest, Effort & Motivation). This high level of overlap in symptom assessment between disorders reflects the fact that the boundaries between mental health disorders are blurry, but also questions the relevance of “disorder-specific” assessment tools. Unlike most physiological disorders where symptoms are used as a guide to diagnosis of an underlying etiology (e.g., fever as a symptom of underlying viral or bacterial infection), mental health disorders are defined purely based on symptom sets and not any underlying physiology or causal factor. Thus, the definition of a disorder as a particular set of symptoms and the subsequent assessment of a “disorder” by a modified set of symptoms results in a circular logic or definitional contradiction. This introduces considerable confusion into the entire system of disorder classification and highlights the challenge of achieving logical consistency in a system of disorders defined on the basis of symptoms rather than etiology or other causal factors.

Assessment tools also varied in terms of the symptom themes that they assessed, with tools for some disorders such as depression and anxiety being more heavily weighted towards asking about emotions and physical aspects of symptoms, while tools for disorders such as ADHD and addiction were more heavily weighted towards questions asking about behavior. This distinction corresponds with the common delineation between internalizing and externalizing spectra (143–145) and raises the question as to whether the delineation solely reflects the clinical manifestation of these disorders, or whether it is also constrained by the corresponding assessment tools. Furthermore, the distribution of symptom themes varied considerably within some disorders. For example, assessments of ASD typically asked about emotional, cognitive and behavioral symptoms to reflect the different manifestations of the disorder (146), but there was considerable heterogeneity in terms of which symptom theme was given the most weighting across different ASD assessments. For example, some assessments gave prominence to behavioral symptoms (e.g., ADI-R), others emphasized cognitive symptoms (all AQ assessment tools) and still others emphasized emotional symptoms (e.g., EQSQ). This bias towards different symptom themes could result in a different clinical picture emerging of the patient’s functioning depending on whether the assessment focused more on their emotional concerns or their behavioral ones. Similarly, when attempting to relate symptom experience against neurophysiological changes or socio-environmental factors, then, for example, assessment tools which highlight the severity of cognitive symptoms may produce different results compared to assessments highlighting behavioral symptoms.

Beyond which symptoms and what aspect of these symptoms were being assessed, the variation also extended to the way that symptoms were being assessed. In particular, the framing of the question in terms of whether it was asking about the frequency, severity, presence, duration, or timing of a symptom was inconsistently applied across assessment tools across disorders. Some disorders emphasized the frequency of symptoms (e.g., for anxiety) while others emphasized the severity of symptoms (e.g., PTSD, schizophrenia, OCD). Similarly, the time period over which symptoms were assessed also varied across disorders. Some disorders were dominated by assessments which considered only recent symptom experience, namely anxiety, bipolar disorder, and depression, while others were assessed by predominantly considering symptoms over the past few months (ADHD, PTSD, OCD) or years (addiction). One caveat to this is that some assessments have different versions which focus on different time periods (e.g., the past month version of CAPS -5 was included in this analysis, but there is also a past week version which was not) and the analysis was therefore somewhat influenced by this. However, the overall consequence of this variability is substantial noise in assessment that both hampers research and the assessment of clinical progress in a patient.

Our results are also in line with previous explorations of the heterogeneity of symptom assessment across different depression tools, where Fried (15) concluded that the “substantial heterogeneity of the depressive syndrome and low overlap among scales may lead to research results idiosyncratic to particular scales used, posing a threat to the replicability and generalizability of depression research”. Given the inconsistency that we have reported here across a much larger group of assessment tools, it warrants the expansion of this conclusion beyond depression to cover the entirety of the mental health field.

### Limitations

In interpreting this result there are several limitations to consider. Firstly, as with any qualitative approach, a degree of judgment and subjectivity is necessary to formulate a systematic coding method. In addition, our methodology involved a subjective grouping of symptoms based on our best judgement, but does not represent the only way, or a hard and fast way, of symptom grouping. A different group of researchers may consolidate symptoms into a different arrangement of symptom categories which could result in a different pattern of results, depending on the number of symptom groupings that they used. Nonetheless, we believe it would be unlikely to change the main finding of vast inconsistency.

Secondly, our analysis of mental health assessment tools was based on the semantic content of the questionnaires and interviews, rather than a patient’s response to those questions. Thus, while the final symptom categories used here are as semantically distinct as possible, they may not be independent from the perspective of underlying etiology in the same way as, for example, high fever and fatigue are semantically distinct but can arise from the same underlying cause.

Another limitation is that for a minority of assessment tools, there were a subset of questions which were included in the assessment, but not included in the assessment scoring. For example, the HCL-32 had 11 unscored questions, the WURS had 36 unscored questions and the YBOCS™ had 60 unscored questions from its checklist section. However, the inclusion of these additional unscored questions generally had only a small impact on the similarity scores as shown in the results. Furthermore, the inclusion of both diagnostic and auxiliary tools, did contribute to some of the differences, especially for the cross-disorder tools, but was not sufficient to explain the picture of heterogeneity across tools.

Finally, we note that our analysis covered 10 disorders selected based on their dominance in the mental health field, excluding less common disorders so as to limit the scope of this study. Furthermore, assessments of age-related disorders such as dementia were not included as they were considered to reflect a different aspect of brain health. It is therefore not possible to say whether the assessment of these disorders align with the results shown here. In addition, it was not possible to include every single assessment available for each of the 10 disorders of interest, and therefore there are others which are not included here. The consequence of this is that our reported scores of symptom similarity are not absolute, but rather generally representative of the field.

### Implications for Mental Health Diagnosis and Research

Historically speaking, mental health diagnosis has been based around disorder-based diagnostic systems of DSM and ICD which map clusters of symptoms onto specific disorders based on phenomenological impressions of psychiatric dysfunction. This classical model of diagnosis has clinical utility in helping psychiatrists and clinical psychologists identify and respond to a diagnostic label corresponding to the array of emotional, cognitive, physical, and behavioral symptoms exhibited by the patient, that in turn guides a path of treatment. It also supports research linking socio-environmental and experiential factors to the onset and trajectory of a disorder. However, the effectiveness of this approach is heavily dependent on having a standardized assessment of symptoms, and clear boundaries between disorders. In contrast, the high degree of symptom overlap between disorders and the inconsistency within disorders shown here, raises concerns about whether disorder prevalence and patient outcomes are simply reflections of the choice of assessment tool. It also has implications for the development and delivery of patient-tailored cognitive-behavioral therapies and pharmacological interventions which target those psychological symptoms, where inconsistencies across symptom assessment may result in suboptimal treatment selection. In addition, such inconsistencies in symptom assessment can introduce challenges when making comparisons across research studies, thus weakening our understanding of psychopathological mechanisms and relevant risk factors. Furthermore, when combined with the comorbid nature of symptom presentation in patients, it exposes the immense challenges faced by psychiatric medicine today.

The heterogeneity of assessment tools also significantly hinders the search for disorder specific and transdiagnostic biomarkers and treatments where results of research studies and clinical trials may depend heavily on the particular bias of the selected assessment tool(s), and where physiological challenges or disturbances do not necessarily align with disorder demarcations. For example, criticism of the current categorical model of disorder classification (147, 148) has led to the emergence of a number of DSM-alternative initiatives that are disorder-independent or spectrum-based. For example, the Research Domain Criteria (RDoC) framework from the National Institute of Mental Health (NIMH) has been developed to provide an arena in which researchers can focus on the neurobiological and neurophysiological markers of psychiatric function and dysfunction, without needing to be aligned to a particular disorder taxonomy and provides a classification structure based around causal mechanisms at multiple levels of genetics, physiology and neuroscience based on data from animals and humans (149–151). In parallel, the Hierarchical Taxonomy of Psychopathology (HiTOP) takes learnings from the dimensional nature of psychopathology and is based on a hierarchical model of mental dysfunction, dominated by externalizing, internalizing and thought disorder spectra (152, 153). It is also affiliated with the development of a single transdiagnostic factor of psychopathology, or “p”, as a way of encapsulating an individual’s propensity to develop all forms of psychopathology (154). However, these models are, as yet, more challenging to operationalize within a clinical setting (155, 156) and the RDoC model has been criticized for specifically downplaying the importance of psychosocial factors (157). The emergence of these alternative transdiagnostic and disorder agnostic approaches are also not immune to inconsistencies in symptom assessment, highlighting the need for tools which have greater standardization in symptom assessment across the mental health landscape, strengthening comparisons across studies and limiting biases and inconsistencies invoked by choosing different sets of assessment tools.

Furthermore, the definition of what actually denotes a mental disorder, and where the boundary between normality and disorder really lies is challenging within such a categorical assessment framework (158). With many “symptoms” such as sadness, anxiousness, and risk-taking representing normal human behaviors, the quantitative boundary between normal and disorder is an important one. Therefore, tools and the research around them, must also strive to understand normality as a way to understand abnormality (159).

### The Potential of Cross-Disorder Assessment

Given the overlap of disorders and the realization that comorbidity is the norm, rather than the exception (160, 161), there is now also increased traction behind the search for transdiagnostic markers of psychiatric dysfunction (11, 162, 163). This is typically done by looking across groups of patients with differential disorder diagnoses, rather than taking a disorder agnostic approach. Disruptions in brain structure and function, cognition and behavior have all been explored from a transdiagnostic perspective, revealing cross-disorder commonalities in the disruption of resting-state functional connectivity (164), cognitive control (165), reward responsivity (166), gray matter volume (167), and social cognition (168). Such work is also supported by evidence showing that some discretely labeled disorders have shared genetic etiology (169–171). Many transdiagnostic studies rely on the DSM system as the diagnostic gold standard (172) despite the heterogeneity in diagnostic symptom criteria both within and across the disorder specific modules of the DSM (173), in line with the results shown here on a larger scale. Furthermore, as we have demonstrated here, none of the available cross-disorder tools provide a comprehensive coverage of the symptoms across all of the 10 disorders of interest. The one with the widest coverage of symptoms was SCID-5-CV at only 86%. Furthermore, the cross-disorder tools themselves were highly variable (especially the auxiliary ones) with an overall average symptom similarity of only 32%. The development of transdiagnostic or disorder agnostic methods that provide a standardized assessment spanning the full breadth of psychiatric symptoms could therefore help advance these endeavors.

While transdiagnostic approaches typically focus on making comparisons at a disorder level, other approaches have focused their efforts at the symptom level, by modelling the interrelationship between co-occurring symptoms and the evolution of symptom covariance using network structures both within disorders and across disorders (174–177). This approach represents individual symptoms as nodes and hubs within a network where the interconnecting lines depict the strength of interrelation between symptoms. The flexibility of this approach, where the networks can adapt and shift both over time to reflect clinical staging, and across individuals, provides an opportunity for delivering a more personalized approach to clinical diagnosis and treatment which is tailored to the individual needs of the patient based on their unique array of symptoms, rather than relying on a “best fit” scenario, as is currently the case. As the network structure is based on reported symptoms, it is therefore also heavily dependent on having standardized assessment tools that can be used to obtain this information.

### Conclusion

In sum, the inevitable evolution of mental health research, diagnosis and treatment into a framework which is closer to underlying biology, transdiagnostic factors or symptom networks, would benefit from a new suite of standardized assessment tools that leave behind historical clustering of symptom criteria and instead provide a disorder agnostic perspective that spans the breadth of mental health function and dysfunction. Like others (178), we join the call for the development of such tools, proposing that attention be given to standardizing not just the suite of symptoms assessed but also to the various aspects of symptom theme, framing and quantification of symptom timing and severity.

## Data Availability Statement

The datasets generated for this study are available on request to the corresponding author. 

## Author Contributions

JN and DH performed the analysis and discussed the results with TT. JN drafted the manuscript. TT and JN revised the manuscript. JN, DH, and TT approved the final version, and agreed to be accountable for all aspects of the work.

## Funding

This work was supported by internal funding from Sapien Labs.

## Conflict of Interest

The authors declare that the research was conducted in the absence of any commercial or financial relationships that could be construed as a potential conflict of interest.

## Footnotes

1
https://www.ncbi.nlm.nih.gov/pubmed/
